# Dust Removal from 3D Point Cloud Data in Mine Plane Areas Based on Orthogonal Total Least Squares Fitting and GA-TELM

**DOI:** 10.1155/2021/9927982

**Published:** 2021-09-13

**Authors:** Jingli Wang, Huiyuan Zhang, Jingxiang Gao, Dong Xiao

**Affiliations:** ^1^School of Transportation Engineering, Shenyang Jianzhu University, Shenyang, China; ^2^College of Information Science and Engineering, Northeastern University, Shenyang 110819, China; ^3^Liaoning Key Laboratory of Intelligent Diagnosis and Safety for Metallurgical Industry, Northeastern University, Shenyang 110819, China; ^4^School of Environment and Spatial Informatics, China University of Mining and Technology, Xuzhou 221116, China

## Abstract

With the further development of the construction of “smart mine,” the establishment of three-dimensional (3D) point cloud models of mines has become very common. However, the truck operation caused the 3D point cloud model of the mining area to contain dust points, and the 3D point cloud model established by the Context Capture modeling software is a hollow structure. The previous point cloud denoising algorithms caused holes in the model. In view of the above problems, this paper proposes the point cloud denoising method based on orthogonal total least squares fitting and two-layer extreme learning machine improved by genetic algorithm (GA-TELM). The steps are to separate dust points and ground points by orthogonal total least squares fitting and use GA-TELM to repair holes. The advantages of the proposed method are listed as follows. First, this method could denoise without generating holes, which solves engineering problems. Second, GA-TELM has a better effect in repairing holes compared with the other methods considered in this paper. Finally, this method starts from actual problems and could be used in mining areas with the same problems. Experimental results demonstrate that it can remove dust spots in the flat area of the mine effectively and ensure the integrity of the model.

## 1. Introduction

Three-dimensional (3D) point cloud has become an important and popular representation of objects in 3D space [1–3]. Multiview stereo-matching techniques can recover 3D models from images or videos, and their typical output format is 3D point clouds [4]. In this paper, a drone is used to photograph the target area, and Context Capture software is used to model and output the 3D point cloud data of the target area. However, it could produce noise points by using the above methods to obtain 3D point cloud data, and the reason is that the dust generated by the truck operation happened to be captured by the drone in the mining area, and the Context Capture software was used to model the photos with dust and the resulting point cloud data contained dust points. Therefore, it is necessary to remove the dust points, which is the same as removing the noise points from the point cloud data.

Rosman et al. [5] proposed a new framework for point cloud denoising through patch cooperative spectrum analysis, which could handle high-level noise gracefully while clearly retaining the surface features of the model. Also, the accuracy and robustness of the algorithm were improved to a certain extent. Mattei and Castrodad [6] proposed moving robust principal component analysis to remove noise, which was effective in denoising point clouds with and without sharp features and had certain advantages compared with traditional denoising methods such as bilateral filtering. Sun et al. [7] proposed an anisotropic point cloud denoising method using L0 minimization. Zheng et al. [8] proposed a point cloud denoising method that could deal with point clouds with sharp features effectively, which retains point cloud features better. The moving least squares (MLS) [9, 10] and local optimal projection (LOP) [11] are two main methods of point cloud denoising. Guennebaud and Gross [9] proposed a new point set surface definition method, which improves the projection stability to a certain extent and can process point cloud data naturally. It is also possible to reliably calculate the average curvature of the surface without cost. Öztireli et al. [10] proposed a new point-based surface definition method to solved some problems of performing approximation in the sense of least squares, which can handle sparse samples, can retain the fine features of the samples, and shows the superiority to a certain extent. Lipman et al. [11] used the LOP operator to approximate the surface of the point cloud data, which works well when the data are noisy. Buades et al. [12] proposed a new algorithm, the nonlocal means (NL-means), based on a nonlocal averaging of all pixels in the image. Dabov et al. [13] proposed the BM3D algorithm to point cloud denoising, which can also remove high noises while ensuring the fine shape features of the sample. Wang et al. [14] and Deschaud and Goulette [15] applied the nonlocal mean denoising method to the point cloud data and used the edge preservation method for point adaptive filtering. Sarkar et al. [16] achieved the desired denoising effect by applying smoothness on the patch dictionary and sparsity on the coefficients and proved the feasibility and advantages through experiments. Wang et al. [17] used k-means clustering algorithm and bilateral filtering denoising algorithm to solve the problem of how to reduce noise points in modeling ancient buildings. The results show that the surface of the point cloud data model is smooth after processing, and the boundary features are maintained well. Zhao [18] aimed at the different scale noise and algorithm time-consuming problems in the process of denoising and smoothing 3D point cloud model data, and the denoising algorithm based on classification idea of point cloud noise was proposed. It divides the noise into two categories including large-scale noise and small-scale noise. Compared with the traditional bilateral filtering, the improved bilateral filtering algorithm is used to smooth the point cloud model data, which increases the calculation rate effectively. In view of the difficulty of removing complex noise in the point cloud data model of cultural relics, a denoising method for point cloud with geometric feature preservation was proposed by Liu et al. [19], which had good denoising effect on cultural relic point cloud data.

**Figure 1:**
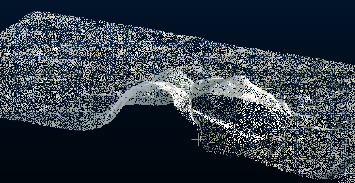
Hollow structure of 3D point cloud model in mining area.

For the hollow model whose point cloud is only concentrated on the surface of the object (Figure 1), the above method applied to the hollow model would cause holes in the model. Therefore, this paper proposes the point cloud denoising method combining orthogonal total least squares fitting and two-layer extreme learning machine improved by the genetic algorithm (GA-TELM). The main steps are to separate the dust points and the ground points with the orthogonal total least squares fitting method and use GA-TELM to repair the holes. The results show that this method can remove noise points without producing holes. The innovations of the paper include the following three points: (1) this method can denoise without generating holes, which solves engineering problems and has engineering significance; (2) compared with other algorithms, GA-TELM has a better effect on repairing holes; and (3) this method also has a better treatment effect for other situations with similar problems.

## 2. Methods

### 2.1. Orthogonal Total Least Squares Fitting Method

The research area is the flat area of the mine whose point cloud data have planar characteristics, and the point cloud data denoising method based on plane fitting can be used. The plane fitting methods based on point cloud data mainly include the least squares method, the eigenvalue method, the total least squares method considering independent variable and dependent variable error [20], and the orthogonal total least squares fitting method [21]. In contrast, the orthogonal total least squares fitting method has higher denoising accuracy and feasibility.

The orthogonal total least squares fitting method is based on the minimum square sum of the orthogonal distances from a point to a plane and takes into account the errors of the dependent variable and the independent variable compared with other plane fitting methods. Therefore, this method can also be used to separate dust points and flat points when the flat area of the mine is uneven. The planar model is as follows:

(1) $$\begin{array}{c} a\left(x-\bar{x}\right)+b\left(y-\bar{y}\right)+c\left(z-\bar{z}\right)=0, \end{array}$$

where *a*, *b*, and *c* are the parameters of the fitted plane to be evaluated, $\bar{x}=\left(1/n\right)\sum_{i=1}^{n}x_{i}$, $\bar{y}=\left(1/n\right)\sum_{i=1}^{n}y_{i}$, $\bar{z}=\left(1/n\right)\sum_{i=1}^{n}z_{i}$, and *n* is the total number of point clouds participating in the fitting plane.

The matrix *M* is constructed, performs eigenvalue decomposition on *M*^*T*^*M*, and takes the eigenvectors corresponding to the smallest eigenvalue as the value of the parameters *a*, *b*, and *c* of the fitting plane. The distance *d*_*i*_ from each point in the target area to the fitting plane is calculated. According to the ranging accuracy *σ*/50, this paper calculates the threshold *δ* of the distance from the point to the fitted plane and judges the relationship between *d*_*i*_ and *δ*. If the *d*_*i*_ < *δ*, the corresponding point is classified as a ground point. Otherwise, it is classified as a dust point.

(2) $$\begin{array}{c} M={\left[\begin{array}{ccc} x_{1}-\bar{x} & y_{1}-\bar{y} & z_{1}-z\\ x_{2}-\bar{x} & y_{2}-\bar{y} & z_{2}-\bar{z}\\ \ldots \ldots & \ldots \ldots & \ldots \ldots \\ x_{n}-\bar{x} & y_{n}-\bar{y} & z_{n}-\bar{z} \end{array}\right]}_{n\times 3}, \end{array}$$

where (*x*_*n*_, *y*_*n*_, *z*_*n*_) represents the *n*th point.

With the aforementioned method, the separation of dust points and ground points is achieved.

### 2.2. GA-TELM

#### 2.2.1. Extreme Learning Machine

Extreme learning machine (ELM) [22–24] is a learning algorithm for single hidden layer feed-forward neural networks, and the only parameter that needs to be set during the training process is the number of nodes in the hidden layer of the network. ELM has a fast learning speed and strong generalization ability and has received wide attention from domestic and international scholars.

*N* samples are (*X*_*i*_, *t*_*i*_), and

(3) $$\begin{array}{c} X_{i}={\left[x_{i1},x_{i2},\ldots ,x_{in}\right]}^{T}\in R^{n},\\ t_{i}={\left[t_{i1},t_{i2},\ldots ,t_{im}\right]}^{T}\in R^{m}. \end{array}$$

There is a single hidden layer neural network with *L* hidden layer nodes, and its output can be expressed as

(4) $$\begin{array}{c} \sum_{i=1}^{L}\beta_{i}g\left(W_{i}\cdot X_{j}+b_{i}\right)=o_{j},\;j=1,2,\ldots ,N, \end{array}$$

where *g*(*x*) is the activation function, *W*_*i*_=[*ω*_*i*1_, *ω*_*i*2_,…,*ω*_*in*_]^*T*^ is the input weight, *β*_*i*_ is the output weight, *b*_*i*_ denotes the bias of the *i*th node of the hidden layer, and *W*_*i*_ · *X*_*j*_ denotes the inner product of *W*_*i*_ and *X*_*j*_.

The goal of single hidden layer neural network learning is to make the output with minimum error, which can be expressed as

(5) $$\begin{array}{c} \sum_{j=1}^{N}\left\|o_{j}-t_{j}\right\|=0,\;j=1,2,\ldots ,N, \end{array}$$

and there are *β*_*i*_, *b*_*i*_, and *W*_*i*_, to make equation (6) true:

(6) $$\begin{array}{c} \sum_{i=1}^{L}\beta_{i}g\left(W_{i}\cdot X_{j}+b_{i}\right)=t_{j},\;j=1,2,\ldots ,N. \end{array}$$

It can be expressed as a matrix:

(7) $$\begin{array}{c} H\beta =T, \end{array}$$

where *H* is the output of the hidden layer node, *β* is the output weight, and *T* is the desired output.

The purpose of network training is to get ${\hat{W}}_{i}$, ${\hat{b}}_{i}$, and ${\hat{\beta }}_{i}$ to make equation (8) true:

(8) $$\begin{array}{c} \left\|H\left({\hat{W}}_{i},{\hat{b}}_{i}\right){\hat{\beta }}_{i}-T\right\|=\underset{W,b,\beta }{\min}\;\left\|H\left(W_{i},b_{i}\right)\beta_{i}-T\right\|,\;i=1,2,\ldots ,L. \end{array}$$

This is equivalent to minimizing the following loss of function:

(9) $$\begin{array}{c} E=\sum_{j=1}^{N}{\left(\sum_{i=1}^{L}\beta_{i}g\left(W_{i}\cdot X_{j}+b_{i}\right)-t_{i}\right)}^{2}. \end{array}$$

*β* can be expressed as follows:

(10) $$\begin{array}{c} \hat{\beta }=H^{+}T. \end{array}$$

Finally, the output of the ELM network is *y*=*Hβ*.

#### 2.2.2. Two-Hidden-Layer Extreme Learning Machine

To improve the accuracy of ELM, Qu et al. [25] added an implicit layer to ELM and proposed a two-hidden-layer extreme learning machine (TELM) [26, 27], which gave a new method to calculate the parameters of the second implicit layer. The simulation experiments show that TELM improves the classification and regression accuracy while the number of nodes in the hidden layer is reduced compared with ELM.

The weights between the first hidden layer and the input layer and the deviation vector of the neurons in the hidden layer of ELM need to be taken randomly. Also, the number of neurons in the hidden layer needs to be set manually.

*W* and *B* of the first hidden layer are set randomly; this paper makes $W_{IE}=\left[\begin{array}{cc} B & W \end{array}\right]$ and $X_{E}={\left[\begin{array}{cc} 1 & X \end{array}\right]}^{T}$, and the output of the first hidden layer can be expressed as follows:

(11) $$\begin{array}{c} H=g\left(W_{IE}X_{E}\right), \end{array}$$

and this paper uses *H* as the final hidden layer output; the output weight *β* of the hidden layer is shown in the following equation:

(12) $$\begin{array}{c} \beta =H^{+}T. \end{array}$$

Then, the expected output *H*_1^*∗*^_ of the second hidden layer is shown in the following equation:

(13) $$\begin{array}{c} H_{1^{*}}=T\beta^{+}. \end{array}$$

The second hidden layer *W*_1_ and *B*_1_ can be calculated from the following equation:

(14) $$\begin{array}{c} W_{HE}=g^{-1}\left(H_{1^{*}}\right)H_{E}^{+}. \end{array}$$

Then, the second hidden layer prediction output *H*_2_ is shown in the following equation:

(15) $$\begin{array}{c} H_{2}=g\left(W_{HE}H_{E}\right). \end{array}$$

**Figure 2:**
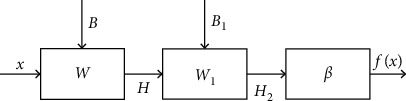
Flowchart of TELM.

So, *β*_new_=*H*_2_^+^*T*. The flowchart of TELM algorithm is shown in Figure 2.

#### 2.2.3. Two-Layer Extreme Learning Machine Improved by the Genetic Algorithm

Because TELM needs to randomly initialize the input weight matrix and deviation vector of the first hidden layer, some weights and deviations may be equal to 0, which means that there will be some invalid nodes in the network. It will reduce the effectiveness and accuracy of the TELM prediction model.

Haber et al. [28] proposed the simple multiobjective cross-entropy method, whose efficiency is corroborated in a real case study represented by the two-objective optimization of the microdrilling process. The proposed strategy performed better than the other methods with higher hyperarea and shorter execution time. Guerra et al. [29] proposed the digital twin-based optimization procedure. The simulation study and the real-time experiments demonstrate the suitability of the digital twin-based optimization procedure and lay the foundations for the implementation of the proposed method at an industrial level. Jia et al. [30] proposed the new optimized radial basis function (RBF) neural network algorithm based on genetic algorithm (GA-RBF), which uses genetic algorithm to optimize the weights and structure; it chooses new ways of hybrid encoding and optimizing simultaneously. Inthachot et al. [31] used artificial neural network (ANN) and genetic algorithm (GA) to predict the trend of Thailand's SET50 index. GA can find better subsets of input variables for importing into ANN, hence enabling more accurate prediction by its efficient feature selection.

GA is the classic optimization algorithm, and scalability is good. It is easy to combine with other algorithms and also the basis for subsequent improvement of other optimization algorithms. In this paper, the classic GA is used to optimize the extreme learning machine to verify the feasibility of the optimization algorithm in such engineering problems. This paper uses GA to optimize the first hidden layer parameters of TELM. The prediction accuracy can be improved to a certain extent.

The pseudocode for the optimization of the weights and threshold parameters of the first implicit layer of the TELM model is as follows :

  In Algorithm 1:
 
*X*: new populations per generation;
 
*P*: training sample input;
 
*T*: training sample output;
 
*P*_test_: test sample input;
 
*T*_test_: test sample output;
  In Algorithm 2:
  hiddennum: number of neurons in the hidden layer;
  NIND: number of individuals in the initial population;
  MAXGEN: maximum number of genetic generations;
 
*px*: crossover probability;
 
*pm*: mutation probability;
  inputnum: number of neurons in the input layer;
  outputnum: number of neurons in the output layer;
 
*w*1num: number of weights from the input layer to the first hidden layer;
 
*w*2num: number of weights from the second hidden layer to the output layer;
 
*w*3num: number of connection weights between two implicit layers;
 
*N*: number of variables to be optimized;

## 3. Experiment

### 3.1. Source of Dataset

LiDAR and UAV tilt photogrammetry are the main methods to generate 3D point cloud models. Both of these technologies are used in actual engineering. In contrast, the cost of LiDAR is higher.

**Figure 3:**
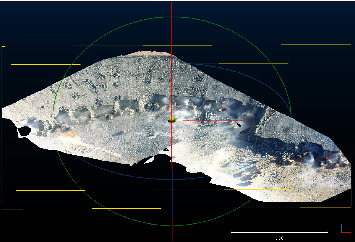
Point cloud model of the mine area.

This paper uses UAV tilt photogrammetry technology to generate the 3D point cloud model. The Cihai open-pit mine in Xinjiang is selected as the experimental research area, and the original image is taken by the DJI Phantom 4 RTK rotor drone. It conducts aerial survey operations at a distance of 120 m above the study area. The flight mode adopts the five-zone “well” flight, and the heading overlap rate and side overlap rate are 80 percent and 70 percent, respectively. The resolution of the obtained image is 20 million pixels. At the same time, the global navigation satellite system (GNSS) receiver was used to collect the 3D coordinates of the control points in the study area. In addition, we use the 3D reconstruction software Context Capture to perform the feature extraction, feature point matching, aerial triangulation, and multiview image matching on the acquired image data. Then, the 3D point cloud model in the study area is generated, as shown in Figure 3. Finally, we use the control point coordinates collected by GNSS to check the 3D point cloud model and judge the accuracy of model construction.

### 3.2. Separation of Point Clouds by Using Orthogonal Total Least Squares Fitting Method

**Figure 4:**
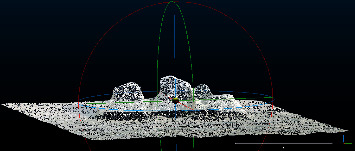
Point cloud model of the experimental area.

**Figure 5:**
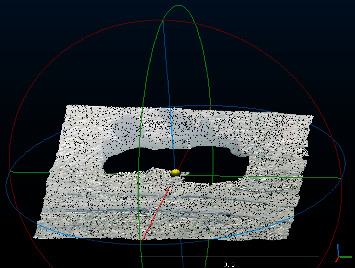
Mode separated by orthogonal total least squares fitting method (ground point).

**Figure 6:**
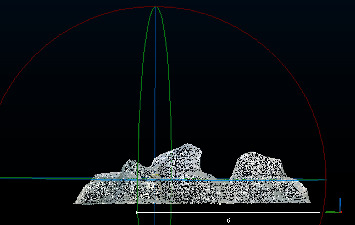
Mode separated by orthogonal total least squares fitting method (dust point).

First, this paper cuts a part of Figure 3 as an experimental model. The experimental model is shown in Figure 4. Then, the orthogonal total least squares fitting method is used to separate the dust point and the plane point. The results of the separation are shown in Figures 5 and 6.  Figure 5 shows a plane point, and Figure 6 shows a dust point. Figure 4 contains 350043 points.  Figure 5 contains 290126 points. Figure 6 contains 59917 points.

### 3.3. Repair of Holes by Using GA-TELM

Ordinary algorithms, such as interpolation, need to create new points by interpolation in the hole. It is necessary to use the machine learning algorithm to process it again to make the new point smoothly connect with the boundary of the hole. This paper uses the dust point separated in the previous step, which can be regarded as a new point generated by interpolation. Therefore, this paper directly uses GA-TELM to complete the repair of the hole.

**Figure 7:**
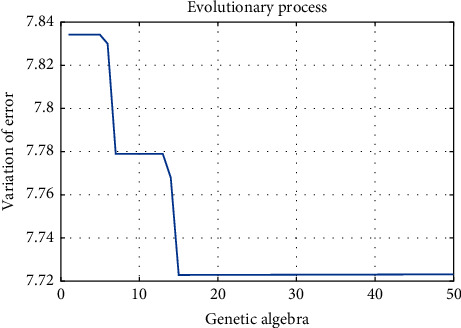
Evolutionary process of GA-TELM.

For the genetic algorithm part of GA-TELM, the relevant parameters set are as follows: NIND is 20, MAXGEN is 50, PRECI is 10, GGAP is 0.95, px is 0.7, and pm is 0.01. The evolutionary process of GA-TELM is shown in Figure 7.

The training set of the 3D point cloud hole repair model based GA-TELM contains the ground points separated by the orthogonal total least squares fitting method (Figure 5), where the *x*, *y* coordinates of the ground points are regarded as the input of the training set and the *z* coordinates of the ground points are regarded as output of the training set. The test set contains the dust points separated by the orthogonal total least squares fitting method (Figure 6), and the *x*, *y* coordinates of the dust points are regarded as test set input.

**Figure 8:**
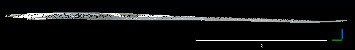
GA-TELM is used to change the dust point of the *z* coordinate.

**Figure 9:**
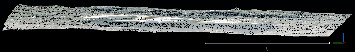
GA-TELM is used to repair the hole in Figure 5.

The output of GA-TELM is used to replace the original *z* coordinate of the dust point, and the result is shown in Figure 8. Figure 8 is Figure 6 after changing the *z* coordinate. Then, Figures 5 and 8 are combined to obtain Figure 9. Figure 9 shows the effect of hole repair.

## 4. Discussion

The computer hardware configuration used in the experiment in this article is Intel Core i5-7500 3.40 GHz processor, 1050Ti graphics card, and 16 GB RAM. In terms of software, the computer uses Windows10 64 bit system, and the simulation environment is Matlab R2018b.

### 4.1. Comparison with Traditional Point Cloud Denoising Methods

Other traditional point cloud denoising methods are to remove noise points from the point cloud. It would reduce the number of points and only apply to solid models. Traditional methods used to solve the actual problems raised in this paper would create holes.  Figure 5 is the result of using least squares plane fitting to denoise, and the traditional point cloud denoising method cannot solve the problems in actual engineering.

### 4.2. Comparison with Different Improved ELM Algorithms

The dataset is regarded as the point cloud data in Figure 5. The dataset is divided into the following two parts: one part is used for network training, accounting for 80 percent, and the other part is used for testing the network, accounting for 20 percent. In this paper, TELM, GA-ELM, and ELM are compared with GA-TELM, and the superiority of GA-TELM is shown more by comparing the time required for the model (Time), the mean square error (MSE), coefficient of determination (*R*^2^), and the test set error (Error).

According to Table 1, GA-TELM has the best MSE, *R*^2^, and test set error among the four networks. *R*^2^ is improved by 2.85 percent than ELM. Although it takes longer time than the other networks, the other three most important aspects have enough advantages. Therefore, this paper uses GA-TELM to perform hole repair. The repair results are shown in Figure 9.

### 4.3. Test of Point Cloud Denoising Method Based on Orthogonal Total Least Squares Fitting and GA-TELM

**Figure 10:**
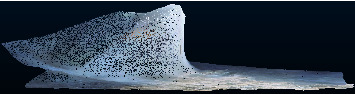
Another flat area containing dust in the mine point cloud model.

**Figure 11:**
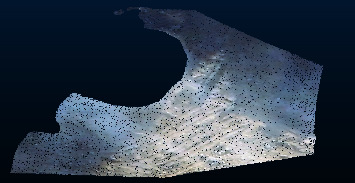
Mode separated by orthogonal total least squares fitting method (ground point).

**Figure 12:**
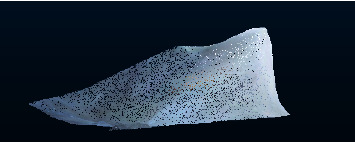
Mode separated by orthogonal total least squares fitting method (ground point).

**Figure 13:**
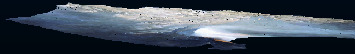
Results of denoising.

This paper selected another point cloud model containing dust points to prove that the denoising method proposed in this paper is suitable for this kind of engineering problem, as shown in Figure 10. First, the least squares plane fitting is used to separate dust points and ground points. Then, GA-TELM is used to repair the holes. The results are shown in Figures 11–13. It can be seen that the denoising algorithm proposed in this paper can solve similar engineering problems.

## 5. Conclusion

This paper proposes the point cloud denoising method based on orthogonal total least squares fitting and GA-TELM, which solves the problems of dust points in the hollow 3D point cloud model. It also has a better treatment effect for other situations with similar problems. The engineering significance of this method is listed as follows: (1) it is used to monitor the engineering volume during the terrain restoration process; (2) it is used for the safety detection of surface settlement in the mining area; and (3) this method verifies the safety of the construction method from semi-direct backfill to complete direct backfill.

## Figures and Tables

**Algorithm 1:**
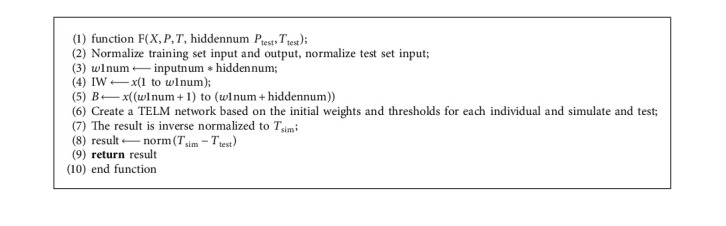
Calculation of the fitness value function.

**Algorithm 2:**
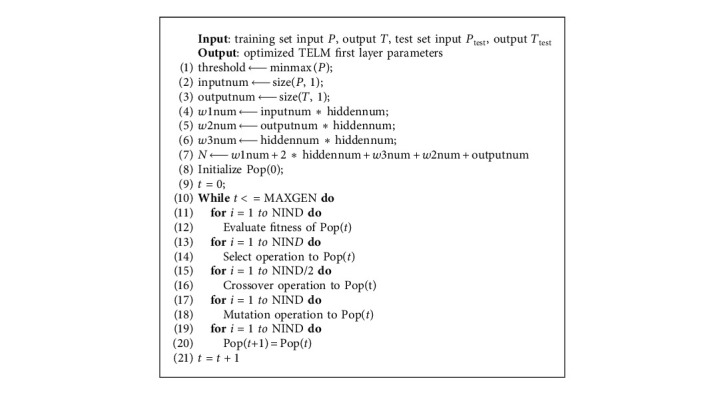
GA-TELM.

**Table 1:** Comparison with different improved ELM algorithms.

Method	Time (s)	MSE	*R* ^2^	Error
GA-TELM	3.978353	0.0010	0.8658	7.7232
TELM	3.674889	0.0011	0.8605	7.8734
GA-ELM	1.252139	0.0011	0.8627	7.8642
ELM	1.016865	0.0013	0.8373	8.5615

## Data Availability

The data used to support the findings of this study are available from the corresponding author upon request.
